# Roles of and cross-talk between ecdysteroid and sesquiterpenoid pathways in embryogenesis of branchiopod crustacean *Daphnia magna*

**DOI:** 10.1371/journal.pone.0239893

**Published:** 2020-10-09

**Authors:** Nikko Adhitama, Yasuhiko Kato, Tomoaki Matsuura, Hajime Watanabe

**Affiliations:** 1 Department of Biotechnology, Graduate School of Engineering, Osaka University, Suita, Osaka, Japan; 2 Biotechnology Global Human Resource Development Program, Division of Advanced Science and Biotechnology, Department of Biotechnology, Graduate School of Engineering, Osaka University, Suita, Osaka, Japan; 3 Frontier Research Base of Global Young Researchers, Graduate School of Engineering, Osaka University, Suita, Osaka, Japan; Lovely Professional University, INDIA

## Abstract

The ecdysteroid and sesquiterpenoid pathways control growth, developmental transition, and embryogenesis in insects. However, the function of orthologous genes and the cross-talk between both pathways remain largely uncharacterized in non-insect arthropods. *Spook* (*Spo*) and *Juvenile hormone acid o-methyltransferase* (*Jhamt*) have been suggested to function as rate-limiting factors in ecdysteroid and sesquiterpenoid biosynthesis, respectively, in insects. In this study, we report on the functions of *Spo* and *Jhamt* and the cross-talk between them in embryos of the branchiopod crustacean *Daphnia magna*. *Spo* expression was activated at the onset of gastrulation, with the depletion of *Spo* transcript by RNAi resulting in developmental arrest at this stage. This phenotype could be partially rescued by supplementation with 20-hydroxyecdysone, indicating that *Spo* may play the same role in ecdysteroid biosynthesis in early embryos, as reported in insects. After hatching, *Spo* expression was repressed, while *Jhamt* expression was activated transiently, despite its silencing during other embryonic stages. *Jhamt* RNAi showed little effect on survival, but shortened the embryonic period. Exposure to the sesquiterpenoid analog Fenoxycarb extended the embryonic period and rescued the *Jhamt* RNAi phenotype, demonstrating a previously unidentified role of sesquiterpenoid in the repression of precocious embryogenesis. Interestingly, the knockdown of *Jhamt* resulted in the derepression of ecdysteroid biosynthesis genes, including *Spo*, similar to regulation during insect hormonal biosynthesis. Sesquiterpenoid signaling via the *Methoprene-tolerant* gene was found to be responsible for the repression of ecdysteroid biosynthesis genes. It upregulated an ortholog of *CYP18a1* that degrades ecdysteroid in insects. These results illuminate the conserved and specific functions of the ecdysteroid and sesquiterpenoid pathways in *Daphnia* embryos. We also infer that the common ancestor of branchiopod crustaceans and insects exhibited antagonism between the two endocrine hormones before their divergence 400 million years ago.

## Introduction

Two endocrine hormones, ecdysteroid and sesquiterpenoid, play important roles in arthropod physiology, development, and phenotypic plasticity [1–5]. Thus, they are believed to have contributed to the evolution of a diverse range of life strategies in arthropods, making them exceptionally abundant and ecologically diverse [6]. The high conservation of genes in the ecdysteroid and sesquiterpenoid biosynthesis pathways across arthropod species (Chelicerata, Myriapoda, Crustacea, and Insecta) is indicative of the vital roles of these two hormones in this clade [7]. The mutagenesis and knockdown of ecdysteroid and sesquiterpenoid pathway genes in insects have demonstrated their functions not only in larva and adults, but also in embryos [8–11].

Ecdysteroid biosynthesis genes in embryos have been widely studied in multiple insect species, including *Drosophila melanogaster* and *Bombyx mori*. Ecdysteroids are supplied maternally into eggs as conjugated ecdysteroid. During early embryogenesis, ecdysteroid-phosphate phosphatase (EPPase) converts the conjugated ecdysteroid into an active form, 20-hydroxyecdysone (20E) [12]. When embryos and larva have *de novo* biosynthesis capability, they synthesize ecdysteroids from cholesterol. The conversion of cholesterol to active ecdysteroids (ecdysteroidogenesis) requires a series of hydroxylation reactions involving multiple enzymes, mostly of the P450 families, including *Noppera-bo* (*Nobo*), *Neverland* (*Nvd*), *Non*-*molting glossy*/*shroud* (*Sro*), *Spook* (*Spo*), *Spookier* (*Spok*), *Cyp6t3*, *Phantom* (*Phm*), *Disembodied* (*Dib*), *Shadow* (*Sad*), and *Shade* (*Shd*) [13]. Genes encoding these enzymes (except *Nvd*, *Cyp6t3*, and *Spok*) are collectively termed as the Halloween genes [13–15]. Mutants of these genes exhibit common phenotypes that result from abnormally low ecdysteroid titers, including a poorly differentiated embryonic cuticle, dorsal closure failure, defective midgut morphogenesis, and eventually embryonic lethality [8, 16, 17].

The sesquiterpenoid hormone is synthesized from acetyl-CoA through the mevalonate pathway. The early steps of the mevalonate pathway to produce farnesyl pyrophosphate (FPP) are conserved among arthropods. The conversion of FPP into its final form of sesquiterpenoid, however, varies among taxa [18, 19]. In chelicerates and some crustaceans, methyl farnesoate (MF) has been reported as the final product of the sesquiterpenoid pathway, while in the majority of insect species, the final product of the sesquiterpenoid pathway is Juvenile Hormone III (JH III) [20, 21]. The rate-limiting reaction of sesquiterpenoid hormone biosynthesis has been thought to be the final conversion into JH III or MF via S-adenosyl-methyltransferase (SAM)-dependent methylation by juvenile hormone acid methyltransferase (Jhamt) [11, 18]. In the hemimetabolous insect *Blattella germanica*, knockdown of *Jhamt* resulted in impaired hatchability [22]. In the holometabolous insect *B*. *mori*, a biallelic mutation of *Jhamt* also reduced hatchability [23]. In both cases, the phenotypic changes produced by modulation of *Jhamt* were not severe and in *B*. *mori*, hatchability could be improved by dechorionating the eggshell. Eventually, mutant embryos were able to grow into larvae, indicating that this hormone is dispensable in the embryonic development of insects.

Recently, cross-talk between these two hormones has been found participate in the regulation of each other’s biosynthesis in *D*. *melanogaster* larvae. Through its response genes, JH reduced the size of prothoracic gland (PG), the portion of ring glands that synthesize ecdysteroid [24]. Meanwhile, the ecdysteroid signaling cascade inhibits the expression of JH biosynthesis genes in the corpora allata (CA) [24]. The antagonism between these two hormones is a key element in the progression of metamorphosis [24]. However, the functions of the biosynthetic genes for these two hormones and the cross-talk between pathways remain to be studied in non-insect arthropod species.

The branchiopod crustacean *Daphnia magna* is not only the most closely related crustacean to insects, but is also the only crustacean with a sequenced genome that can be easily manipulated [25]. Its draft genome sequence and transcriptome data are publicly available [26–28]; moreover, gene manipulation techniques, including RNAi, TALEN, and CRISPR/Cas9, have been established in this organism [29–35]. In a nutrient rich environment, *D*. *magna* produces parthenogenetic eggs and increases its population asexually. Its embryos show typical direct development [36]. Hatching of *D*. *magna* embryos occurs when appendage segmentation ends [37]. In insects, this stage seems to be consistent with timing of blastokinesis [38]. When the hatched embryos become juveniles, they molt to attain their complete morphology, with structures including setae of the second antennae and a tail spine. The hatched embryo subsists on its own yolk supply, similarly to later stages of the hemimetabolan embryo, or pronymph [39]. Previous reports have identified the presence of homologs of genes involved in ecdysteroid and sesquiterpenoid biosynthesis and signaling in *Daphnia* genomes [40–43]. The ecdysteroid biosynthesis pathway had been partly analyzed by the knockdown of *Neverland* (*Nvd*) and *Shade* (*Shd*) [44, 45], suggesting that both genes play roles in *de novo* ecdysteroid synthesis during embryogenesis. Functional analysis related to the sesquiterpenoid biosynthesis genes and signaling has also been conducted. The analysis of Jhamt in *D*. *pulex* confirmed its role in MF biosynthesis and suggested that it has a role in male offspring production in adults [43].

In this study, we analyzed the function of *Spo* and *Jhamt* during the progression of *D*. *magna* embryogenesis. We confirmed the role of *Spo* in ecdysteroid biosynthesis in early embryos. We present evidence of the potential function of *Jhamt* and sesquiterpenoid in the regulation of embryonic timing of *D*. *magna*. Moreover, *Jhamt*/sesquiterpenoid controls genes that are involved in ecdysteroid metabolism.

## Results

### Cloning and characterization of *Spo* and *Jhamt*

**Fig 1:**
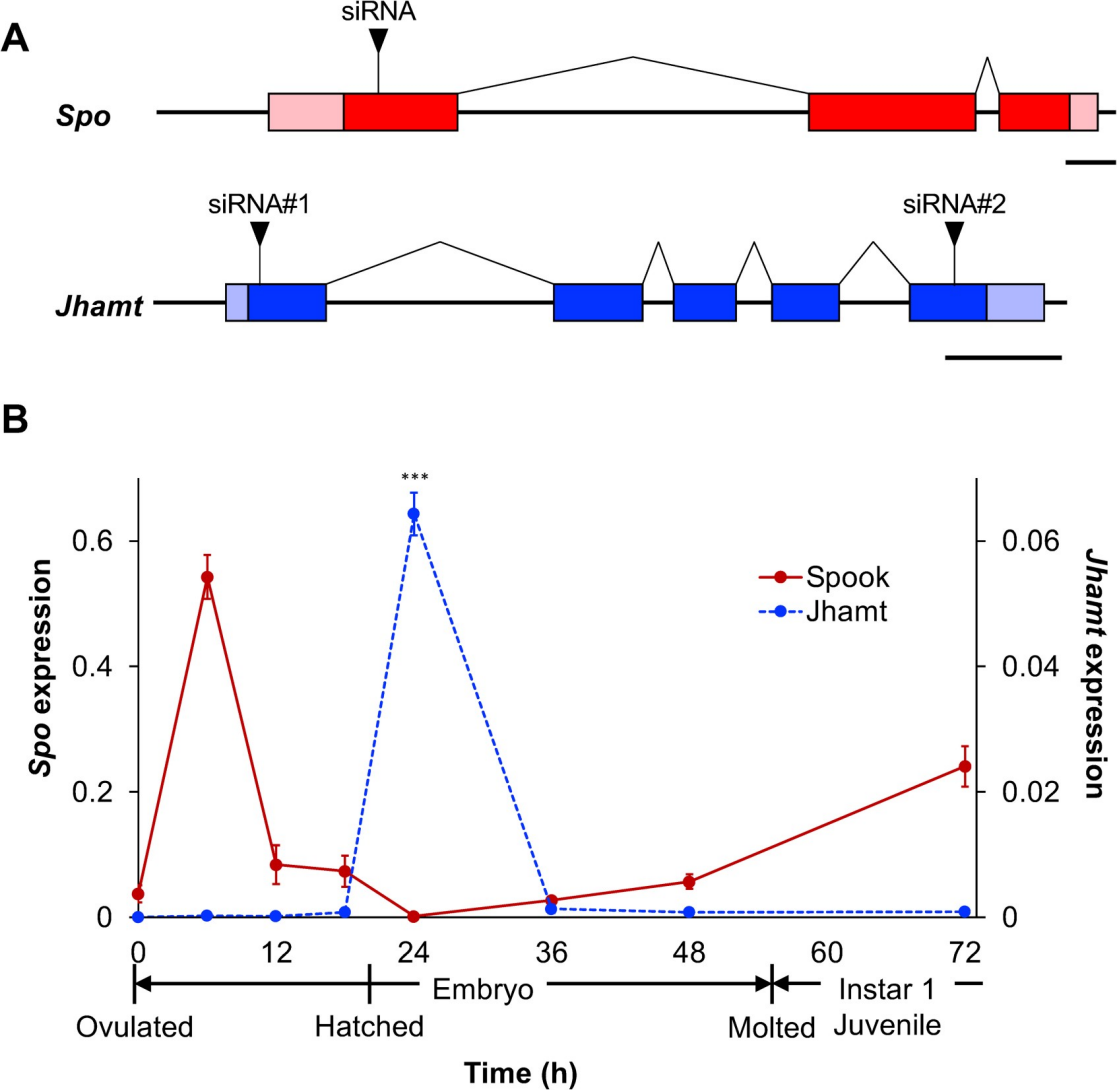
Gene structures and temporal expression patterns of *Spo* and *Jhamt* during embryogenesis. (A) Schematics of *Spo* (red) and *Jhamt* (blue) genes. Coding sequences (CDSs) are shown in dark-colored boxes and untranslated regions (UTRs) are indicated in light-colored boxes. siRNA target sites are denoted by black triangles. Scale bar, 250 nt. (B) Expression of *Spo* (red) and *Jhamt* (blue) during embryogenesis quantified by qRT-PCR. Timing of hatching and molting to instar 1 juveniles are shown under the x-axis. Expression levels are normalized to those of the reference gene ribosomal *L32*. Values are means. Error bars represent SD (N = 3). ***p<0.001 (Student’s *t*-test).

We performed a TBLASTN search using the amino-acid sequences of *Spo* and *Jhamt* obtained from several insect species against the *D*. *magna* genome database (http://arthropods.eugenes.org/EvidentialGene/daphnia/daphnia_magna/). One ortholog of *Spo* was located at scaffold 02011 in the database. The full sequence of the *Spo* cDNA was determined using 5′ and 3′ RACE. Comparing the *Spo* cDNA to genome sequences revealed that it contains 3 exons and 2 introns, spanning 4,191 bp in the genome (Fig 1A). The *Spo* coding sequence (CDS) was found to encode a 591-residue protein with a molecular weight of 66 kDa (S1A Fig). Multiple alignment with fruit fly (*D*. *melanogaster*), silkworm (*B*. *mori*), and red flour beetle (*Tribolium castaneum*) orthologs revealed that *D*. *magna Spo* contains typical CYP450 characteristic domains: Pro/Gly cluster, WxxxR, ExxR, PERF motif, and heme loop (S1B Fig).

The *Jhamt* gene was found at scaffold 00915. We sequenced a full-length the *Jhamt* cDNA, revealing that this gene spans 1,767 bp and consists of 5 exons and 4 introns (Fig 1A). It encodes a protein of 270 residues with a molecular weight of 31 kDa (S2A Fig). We identified a putative S-adenosyl-L-methionine (SAM) domain located in the N-terminus that is characteristic of the methyltransferase protein family.

### *Spo* is essential for the progression of early embryogenesis

We first measured *Spo* gene expression levels using qRT-PCR. Expression peaked at 6 h after ovulation (hao) before abruptly dropping to its lowest level shortly after hatching at 24 hao. Expression then steadily increased throughout the remainder of embryogenesis (Fig 1B).

**Fig 2:**
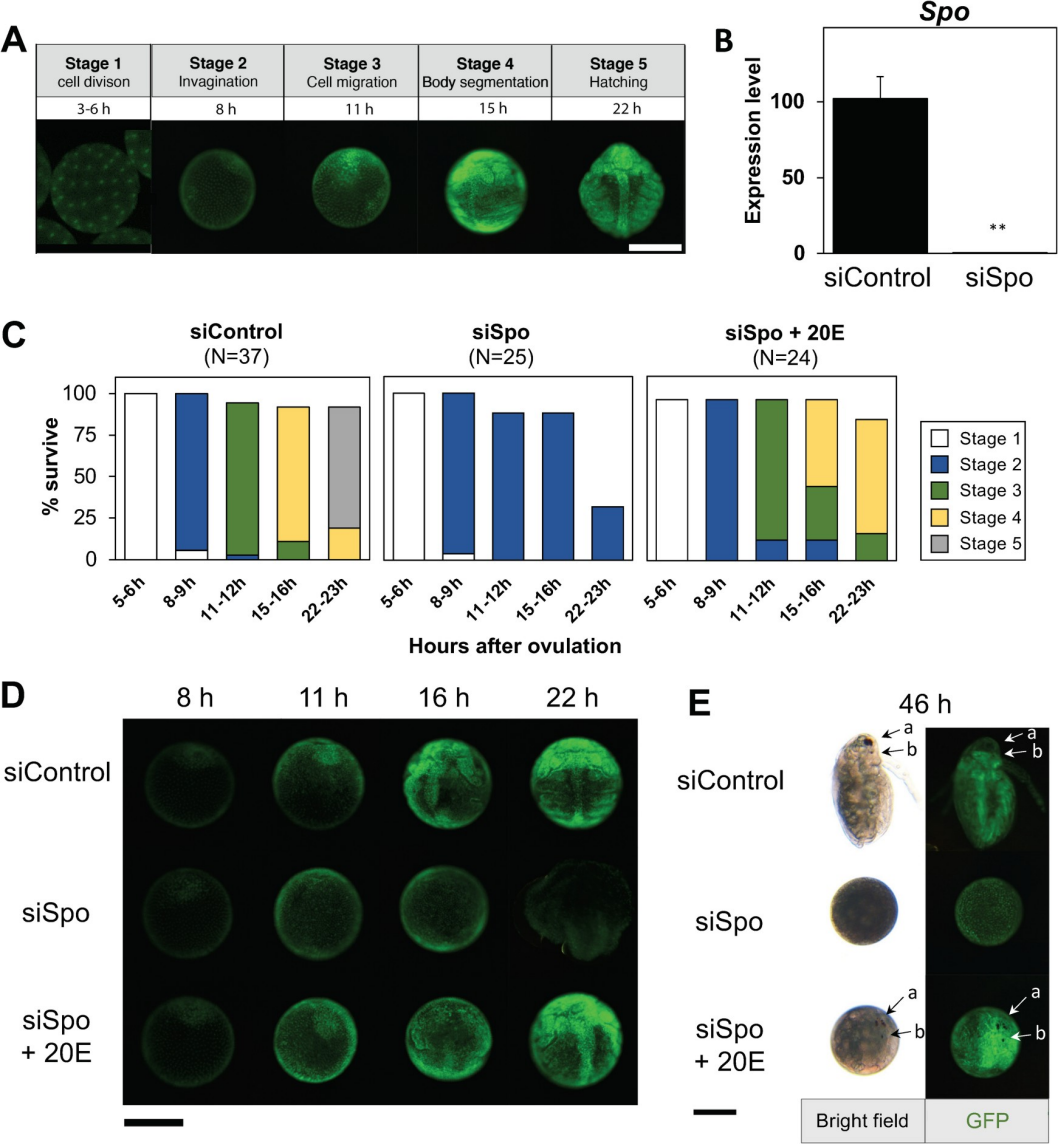
*Spo* is essential during early embryogenesis in *Daphnia magna*. (A) Developmental stages of *D*. *mag*na during early embryogenesis at 23°C as observed by GFP fluorescence in an H2B-GFP transgenic line. (B) Expression levels of *Spo* in siControl and siSpo embryos at 6 h assessed by qRT-PCR. Values are means. Error bars represent SD (N = 3). **p<0.01 (Student’s *t*-test). (C) Developmental progression and survival rate of siControl and siSpo with or without exposure to 1 μM 20-hydroxyecdysone (20E). Bars denote the developmental stages described in (A): stage 1 (white), stage 2 (blue), stage 3 (green), stage 4 (yellow), stage 5 (gray). (D) Representative images of embryos injected with siControl, siSpo, and siSpo rescued by 20E. Embryos were imaged by GFP fluorescence. Representative photographs from different stages are combined into a single image. (E) Retardation of embryo developmental progression. Compound eye (a) and naupliar eye (b) development observed at 46 h after ovulation. Scale bar, 200 μm.

To investigate the role of *Spo*, we performed loss of function analysis using RNA interference (RNAi). To easily track its developmental progression in the embryo stage, we utilized a previously established transgenic *Daphnia* that ubiquitously expresses an H2B-GFP fusion protein [30]. We determined a staging parameter as previously described [30, 37] (Fig 2A). Cleavage and blastula formation occurred during the first 6 hao (Stage 1). Shortly after, the appearance of an invagination pit was indicative of gastrulation (Stage 2). Following gastrulation, cell mass migration proceeded, with the formation of three germ layers (Stage 3). At around 15 hao, the formation of thoracic appendages and a pair of secondary antennae was observed (Stage 4). Hatching occurred around 22 hao (Stage 5).

We initially confirmed that siRNA-*Spo* significantly reduced *Spo* transcripts at 6 hao (Fig 2B). Embryos injected with siRNA-*Spo* showed no developmental differences relative to siRNA-ctrl-injected embryos until stage 2, during cleavage and cell mass migration (Fig 2C and 2D). Subsequently, all of the injected embryos showed a retardation in development and failed to proceed beyond stage 2 (Fig 2C and 2D; siSpo), in contrast to control embryos (Fig 2C and 2D; siControl). This developmental arrest was consistent with the *EcR* RNAi phenotype observed in our previous study [46].

To demonstrate the role of *Spo* in ecdysteroid biosynthesis in *D*. *magna* embryogenesis, we attempted to rescue the phenotype of *Spo* RNAi embryos by supplementing the culture medium with 20E. As expected, 20E-exposed *Spo* RNAi embryos progressed beyond stage 2 (Fig 2C and 2D; siSpo+20E). Around 70% of rescued embryos progressed to stage 4 (Fig 2C; siSpo+20E). Of the rescued embryos, 10% developed further and formed naupliar and compound eyes after 46 hao (Fig 2E) but did not become juveniles. These results demonstrate that *Spo* is essential for early embryogenesis, likely via ecdysteroid biosynthesis.

### *Jhamt* prevents precocious embryonic development

**Fig 3:**
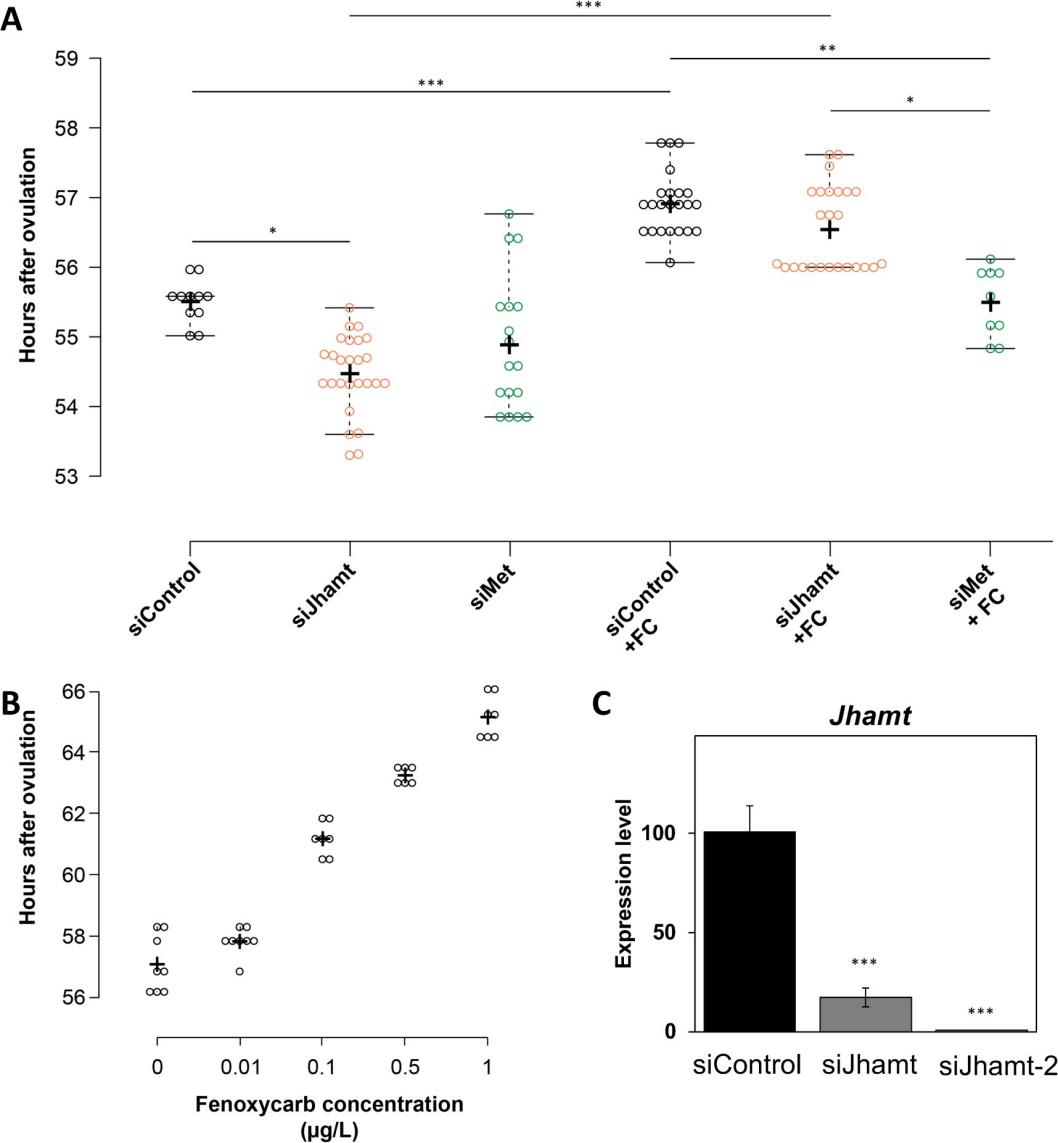
*Jhamt* knockdown shortens *Daphnia magna* embryonic period. (A) Embryonic periods (hours after ovulation, or hao) of siControl, siJhamt, and siMet embryos with or without fenoxycarb. *p<0.05; **p<0.01; ***p<0.001 (Kruskal-Wallis followed by Dunn post-hoc test; p-values adjusted using the Benjamini-Hochberg procedure). (B) Embryonic period of embryos exposed to different concentrations of fenoxycarb. (C) Expression levels of *Jhamt* in 24 h embryos injected with siControl and siJhamt as measured by qRT-PCR. Values are means. Error bars represent SD (N = 3). **p<0.01 (Student’s ***t***-test).

In contrast to *Spo*, *Jhamt* mRNA was rarely detected during embryogenesis, with the exception of a significant expression surge at 24 hao (Fig 1B). To determine the function of *Jhamt* during embryogenesis, we injected eggs with siRNA-*Jhamt*. However, no significant difference in hatching or survival was observed in Jhamt RNAi embryos, suggesting that *Jhamt* loss of function has little effect on embryo survival (Table 1). Because sesquiterpenoids are known as “*status quo”* hormones in insects, we also examined the timing of molting after the end of embryogenesis. siRNA-*Jhamt*-injected embryos showed a significant reduction during the embryonic period compared with the control (Fig 3A; siJhamt), suggesting that sesquiterpenoid biosynthesis represses the precocious transition of embryos into the juvenile stage.

**Table 1:** RNAi inhibition of sesquiterpenoid pathway genes.

siRNA	Total injected eggs	Relative hatchability (%)	Relative survivability (%)
siControl	104	100± 13	100± 19
siJhamt	133	105 ± 3^a^	102 ± 11^b^
siMet	95	105 ± 5^c^	86 ± 4 ^d^

Values are means ± SD.

^a^p = 0.30

^b^p = 0.43

^c^p = 0.29

^d^p = 0.84 (Welch’s *t*-test).

To elucidate the regulatory role of sesquiterpenoid signaling in embryos, we silenced *Met*, which codes for the sesquiterpenoid receptor. Consistent with siRNA-*Jhamt*, *Met* RNAi had little effect on embryo survivability (Table 1). There were similarities with the *Jhamt* RNAi embryos during embryogenesis, although there was no significant difference between the control and *Met* RNAi treatment (Fig 3A; siMet). We also examined the effect of exposure to the sesquiterpenoid analog Fenoxycarb (FC), on this phenotype. FC increased the length of the embryonic period in both control and *Jhamt* siRNA-injected embryos (Fig 3A; siControl+FC, siJhamt+FC) in a FC concentration-dependent manner (Fig 3B). In contrast, FC treatment of siRNA-*Met* injected embryos did not significantly elongate the embryonic period (Fig 3A; siMet+FC), possibly due to the silencing of sesquiterpenoid signaling. A reduction in the expression level of the *Jhamt* transcript was observed upon siRNA-*Jhamt* injection (Fig 3C). These results suggest that *Jhamt* plays an important role in sesquiterpenoid biosynthesis and the regulation of the embryonic period.

### Sesquiterpenoid signaling regulates the expression of ecdysteroid metabolism genes in hatched embryos

**Fig 4:**
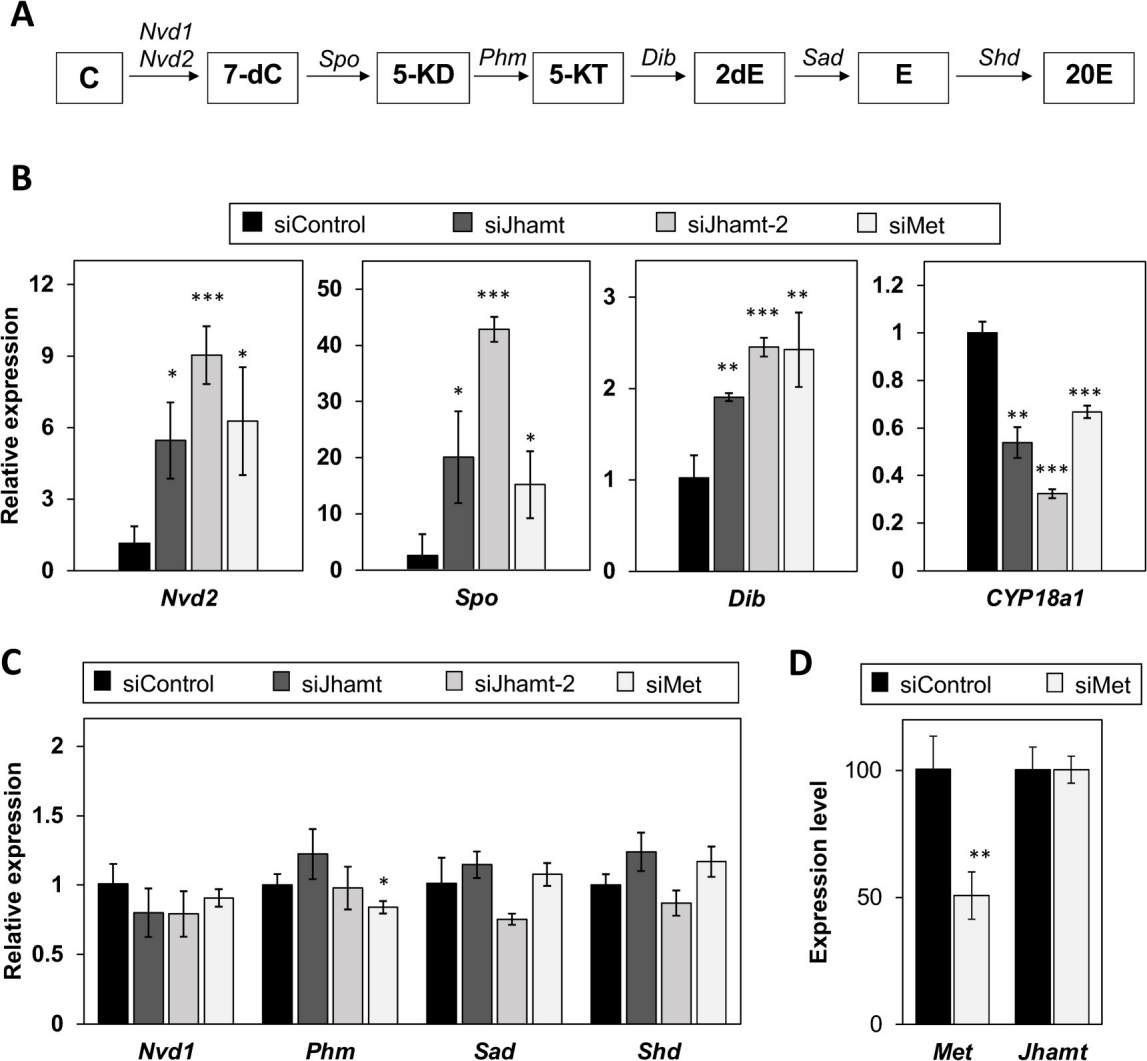
*Jhamt* knockdown upregulates expression of ecdysteroid biosynthesis genes at 24 hao. (A) Predicted ecdysteroid biosynthesis pathway in *Daphnia magna*. C: cholesterol; 7-dC: 7-dehydrocholesterol; 5-KD: 5β-ketodiol; 5-KT: 5β-ketotriol; 2dE: 2-deoxyecdysone; E: ecdysone; 20E: 20-hydroxyecdysone. Nvd: Neverland; Spo: Spook; Phm: Phantom; Dib: Disembodied; Sad: Shadow; Shd: Shade. (B) Expression of *Nvd2*, *Spo*, *Dib*, and *Cyp18a1* at 24 h in siControl, siJhamt and siMet embryos at 24 h after siRNAt as measured by qRT-PCR. (C) Expression levels of other ecdysteroidogenesis genes in siControl, siJhamt, and siMet embryos. Expression levels are normalized to those of ribosomal *L32*, then shown relative to expression levels of siRNA-ctrl. Values are means. Error bars represent SD (N = 3). *p<0.05; **p<0.01; ***p<0.001 (Student’s *t*-test).

Unexpectedly, significant upregulation of *Jhamt* expression was found to be coincided with low levels of *Spo* transcript expression in hatched embryos at 24 hao (Fig 1B). This antagonistic pattern of *Spo* and *Jhamt* expression suggested the hypothesis of cross-talk between ecdysteroid and sesquiterpenoid biosynthesis at this stage. To investigate this hypothesis, we measured *Spo* expression in *Jhamt* RNAi embryos, observing a significant increase at 24 hao after siRNA injection (Fig 4B). We confirmed the specificity of this *Spo* activation using a different *Jhamt*-targeting siRNA, siRNA-*Jhamt*-2 (Fig 4B). We analyzed the expression of other genes functioning in ecdysteroidogenesis pathways in insects (Fig 4A, 4B and 4C) and found that *Nvd2* and *Dib* were upregulated (Fig 4B and 4C). Furthermore, we measured the expression of *Cyp18a1*, which is responsible for the degradation of ecdysteroid [45, 47]. In contrast to ecdysteroidogenesis genes, *Cyp18a1* expression was reduced by *Jhamt* RNAi (Fig 4D).

We also examined the effect of *Met* RNAi on genes related to ecdysteroid metabolism. Consistent with the effect of siRNA-*Jhamt* injection, mRNA levels of *Spo*, *Nvd2*, and *Dib* (Fig 4B) were increased, while *Cyp18a1* expression was decreased, with no effect on the other genes (Fig 4C). The level of *Jhamt* mRNA expression was not affected by *Met* knockdown (Fig 4D), suggesting that sesquiterpenoid signal transduction regulates ecdysteroid biosynthesis and degradation, respectively. Taken together, our results suggest cross-talk between the sesquiterpenoid and ecdysteroid metabolic pathways in hatched embryos.

## Discussion

The endocrine hormones ecdysteroid and sesquiterpenoid are known to regulate developmental transition, growth duration, and embryogenesis in insects. Recently, genome sequencing studies have revealed that genes related to ecdysteroid and sesquiterpenoid biosynthesis are highly conserved, indicating the importance of these two hormones across arthropod species [7]. However, their functions have not yet been elucidated in non-insect arthropods, which has prevented a deeper understanding the functional similarity and diversity of genes related to these hormonal pathways. In this study, we reported on the functional analysis of potential rate-limiting genes *Spo* and *Jhamt*, and cross-talk between ecdysteroid and sesquiterpenoid biosynthesis pathways in a branchiopod crustacean. A combination of RNAi and hormone administration indicated that *Spo* and *Jhamt* play roles in ecdysteroidogenesis and sesquiterpenoid biosynthesis, respectively, during embryogenesis. We also found evidence of cross-talk between the ecdysteroid and sesquiterpenoid pathways.

The expression pattern and functional analysis of *Spo* confirmed its indispensable role in ecdysteroidogenesis during *D*. *magna* embryogenesis. The *Spo* transcript was highly expressed during early embryonic stages and peaked at 6 hpo, which coincides with the onset of gastrulation [30], indicating that ecdysteroid is required for embryonic cell differentiation. *Spo* expression in the *D*. *magna* embryo showed a similar expression pattern to that in the early embryo of *D*. *melanogaster*, where *Spo* has been found to be expressed during pre-cellular blastoderm stage, prior to gastrulation [17]. In *D*. *magna*, the RNAi-mediated knockdown of *Spo* resulted in embryonic arrest during gastrulation. This phenotype is consistent with Halloween gene mutants previously reported in insects [16, 17, 48, 49], suggesting its conserved role in embryonic ecdysteroid biosynthesis. Interestingly, Spo RNAi embryos showed no developmental differences before 8 hpo, which includes the processes cleavage and early gastrula formation (Fig 2B and 2C). This suggests that embryos utilized maternally supplied ecdysteroid conjugates instead of performing *de novo* ecdysteroidogenesis before gastrulation. Indeed, in a previous study, we found that the expression of *EPPase* dropped sharply after 6 hpo, which was consistent with the activation of *Spo* [50]. This pattern suggests that *Spo* begins to take over ecdysteroidogenesis after the depletion of maternally supplied ecdysteroid or EPPase. *Spo* RNAi embryos supplemented with 20E resumed development, although hatching was never achieved (Fig 2C, 2D and 2E). In *Drosophila*, the *Spo* mutant phenotype could be completely rescued by the administration of 20E, eventually progressing to adulthood [17]. This is possible because in *Drosophila* and other dipteran species, *Spo* is only expressed during embryogenesis, while another paralog, *Spookier* (*Spok*), takes over ecdysteroidogenesis in later stages [17, 51]. Since *D*. *magna* only has one ortholog of *Spo*, 20E supplementation may not be sufficient to completely rescue embryogenesis.

The knockout and knockdown of *Jhamt* are known to affect development in insects. In a previous study, *Jhamt* and *Met* RNAi resulted in impaired hatchability of embryos in the hemimetabolous insect *Blattella Germanica* [22]. In the holometabolous insect *B*. *mori*, *Jhamt* knockout was performed using TALENs. The resulting biallelic mutation did not have any effect on embryonic development in homozygous *Jhamt* mutants generated by crossing heterozygous adults, but led to a decreased embryo hatching rate [23]. These results suggest that *Jhamt* plays a role in hatchability in insects. In *D*. *magna*, neither *Jhamt* nor *Met* RNAi severely affected hatching of embryos or the survival of juveniles, suggesting that sesquiterpenoid signaling in *D*. *magna* embryogenesis is less important than it is in insect embryos. This may be due to the short duration of *Jhamt* expression in the *Daphnia* mid-embryonic stage compared with a longer duration from mid- to late embryogenesis in *B*. *germanica* and *B*. *mori*. The *Met* RNAi phenotype in this study was different from that observed in a previous study, in which embryos injection with long, double-stranded RNA targeting *Met* arrested embryogenesis [42]. Although the reason for this contradiction is unclear, this embryonic-lethal phenotype was neither evaluated by injecting another non-overlapping *Met* dsRNA nor by a rescue experiment. In *D*. *pulex*, *Jhamt* was reported to be expressed during the juvenile and adult molting cycles [43, 52]. Moreover, in *Daphnia*, sesquiterpenoid has been found to regulate phenotypic plasticity in predator defense [5, 53, 54] and environmental sex determination [55, 56]. Therefore, the role(s) of sesquiterpenoid in *Daphnia* may change in later life stages, as observed in insects [57]. To disrupt *Jhamt* function completely, we should generate and cross heterozygous *D*. *magna* mutants. However, the hatchability and genetic stability of embryos produced by sexual reproduction is low due to the accumulation of deleterious homozygous mutations, as this species typically undergoes parthenogenetic reproduction, preventing us from more precisely evaluating the function of *Jhamt* in hatching. To overcome this problem, a conditional knockout method will need to be developed in *D*. *magna* in the future.

Our results indicated that *Jhamt* and sesquiterpenoid signaling via *Met* play a role in the repression of precocious development, as their knockdown reduced the duration of the embryonic period, while supplementation with a sesquiterpenoid analog elongated embryogenesis. This phenotype is in concordance with the well-known effect of sesquiterpenoid, a “*status quo”* action. In contrast, a *B*. *mori Jhamt* mutant showed an increased embryonic period, while a sesquiterpenoid analog-exposed cricket showed precocious embryonic development, demonstrating that *D*. *magna* may have uniquely co-opted sesquiterpenoid signaling for the timing of embryogenesis.

A possible advantage of the repression of precocious embryonic development may be the synchronization of embryogenesis with ovarian development. In *D*. *magna*, once eggs are laid inside the mother’s brood chamber, the embryos develop in this chamber until their first juvenile instar stage. Interestingly, embryogenesis is almost perfectly synchronized with the maturation of the mother’s ovaries. Shortly after the release of the first instar juveniles from the brood chamber, the mother molts and lays the next batch of eggs. A shortening of embryonic development leads to the earlier release of juveniles from the brood chamber. In contrast, when embryonic development is delayed and embryos are kept longer, they will be released together with the mother’s carapace, possibly increasing the risk of predation.

**Fig 5:**
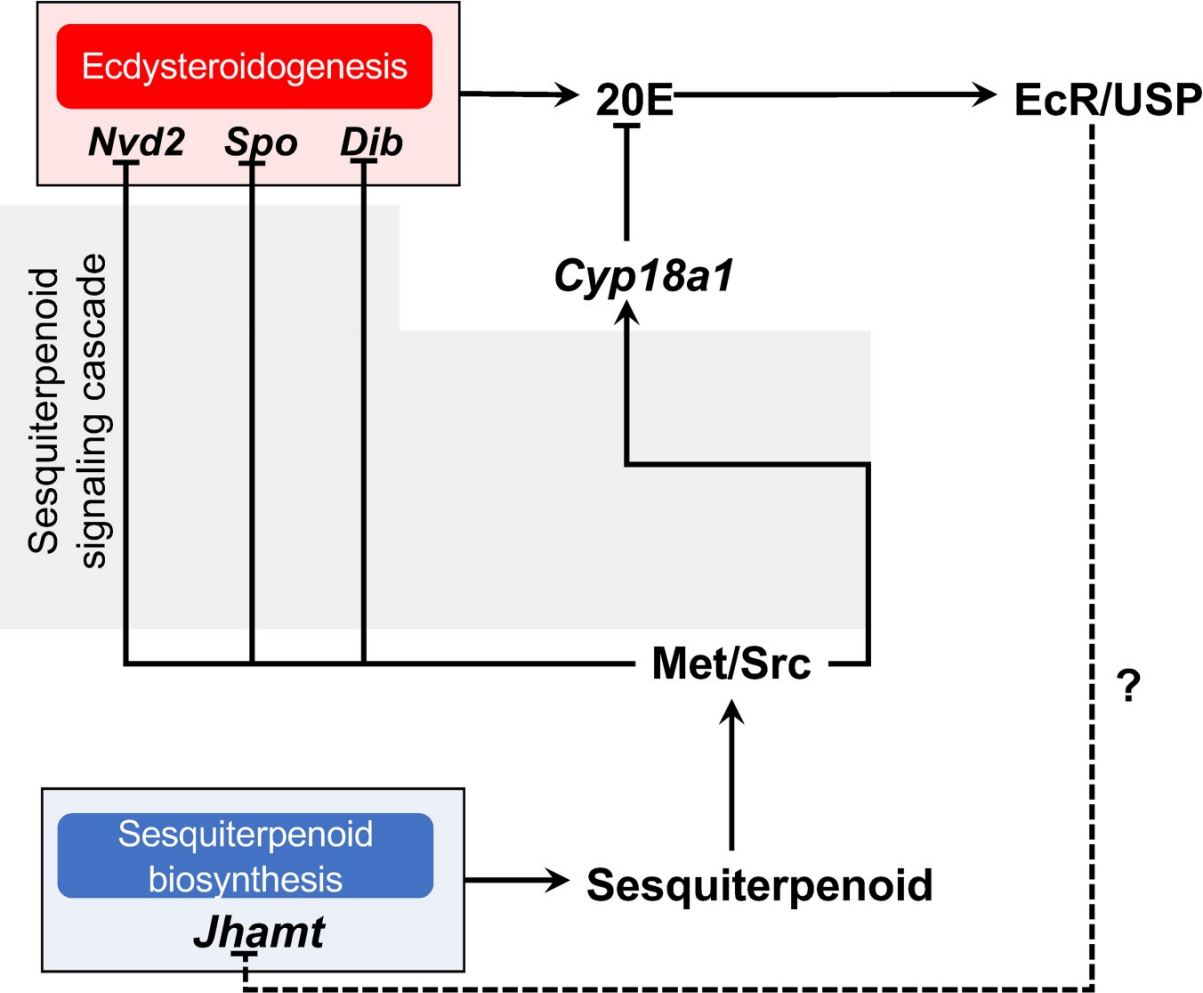
Schematic diagram of proposed ecdysteroid and sesquiterpenoid cross-talk in *Daphnia magna* embryo. Solid lines indicate direct interactions whereas dashed line indicates possible interaction. Sesquiterpenoid through sesquiterpenoid signaling cascade downregulates ecdysteroidogenesis gene expression, hence suggesting antagonistic relationship between sesquiterpenoid and ecdysteroid.

The antagonistic relationship between ecdysteroid and sesquiterpenoid biosynthesis has been previously reported in insects [24, 58]. A recent study on the *Drosophila* ring gland showed that JH induces *Kr-h1* expression, whose gene product inhibits ecdysteroid biosynthesis by suppressing EcR/USP and ecdysone-induced early transcription factors (TFs) (Broad complex, E75, and E73), resulting in reduced PG size and subsequently the inhibition of metamorphosis. *Vice versa*, 20E via EcR/USP action suppress the JH biosynthesis genes *Jhamt* and *HMG-CoA reductase* (*Hmgcr*) in CA, thereby lowering the JH level and allowing the larva to proceed to metamorphosis [24]. In this study, we provide evidence of antagonism between the ecdysteroid and sesquiterpenoid biosynthesis mechanisms during *D*. *magna* embryogenesis (Fig 5). Interestingly, we also observed a significant downregulation of *Cyp18a1*, which is responsible for ecdysteroid inactivation [45, 47] (Fig 4B). To the best of our knowledge, this study is the first to demonstrate a correlation between ecdysteroid inactivation and the antagonistic action of sesquiterpenoid, indicating that sesquiterpenoid plays a broader role in the regulation of ecdysteroid metabolism. The regulation of ecdysteroidogenesis by sesquiterpenoid is likely to be orchestrated by a sesquiterpenoid signaling cascade, since the *Met* knockdown caused similar ecdysteroid biosynthesis gene upregulation. It will be important to clarify which downstream components of sesquiterpenoid signaling inhibit ecdysteroid biosynthesis genes in *Daphnia*. In contrast, in this study, we were unable to determine the role of ecdysteroid in the regulation of sesquiterpenoid biosynthesis or metabolism in *D*. *magna* since the currently available approaches for the impairment of ecdysteroid signaling or biosynthesis resulted in embryonic lethality during early embryogenesis [45, 46]. Nevertheless, our results suggest that ecdysteroid and sesquiterpenoid hormonal cross-talk was established before the evolutionary divergence of *Daphnia* from insects over 400 million years ago.

This study demonstrates the roles and cross-talk of ecdysteroid and sesquiterpenoid biosynthesis during embryogenesis in a branchiopod crustacean, *D*. *magna*. Similar to insects, ecdysteroidogenesis is necessary for early embryogenesis in *D*. *manga*. Sesquiterpenoids regulate the expression of ecdysteroid metabolism genes, including *Spo*, and repress precocious embryonic development. We anticipate that this work will contribute for furthering our understanding of the evolution and diversity of arthropod endocrinology.

## Materials and methods

### Daphnia strains and transgenic line

All daphnids were raised under the following conditions: 80 neonates (under 24 h) were transferred to 5 L medium and cultured at 22–24°C, under a constant light/dark photoperiod of 16 h/8 h. Artificial Daphnia Medium (ADaM) was used as the culture medium and prepared using reverse osmosis (RO) water, as reported previously [59]. Daphnids were fed daily with a 100 μL suspension of 8×10^9^ cells/mL *Chlorella vulgaris* (Oitamedakabiyori, Oita, Japan) and 15 μL suspension of 0.15 g/mL baker’s yeast (Marusan Pantry, Ehime, Japan) during the first week. Upon reaching reproductive age, their offspring were removed once per day and fed daily with a 200 μL suspension of 8×10^9^ cells/mL chlorella and 30 μL suspension of 0.15 g/mL baker's yeast.

The wildtype strain (NIES clone) was obtained from the National Institute of Environmental Studies (NIES, Tsukuba, Japan). We utilized previously established transgenic *Daphnia* that express a H2B-GFP fusion protein under *Daphnia magna elongation factor 1-α* (*Ef1α*) promoter/enhancer [30] to visualize the progression of embryogenesis.

### RNA isolation and purification

*Daphnia* (adults or embryos) were collected in 2-mL tubes, immediately frozen in liquid nitrogen, and homogenized using a MicroSmash MS-100 machine (TOMY, Tokyo, Japan) in the presence of Sepasol-RNA I reagent (Nacalai Tesque, Kyoto, Japan), according to the manufacturer’s instructions. Extracted total RNA was further purified using phenol-chloroform extraction and ethanol precipitation. Purified total RNA was dissolved in RNase free water (Invitrogen, Carlsbad, USA) and stored at –80°C until further use.

### Cloning and sequencing of Spo and Jhamt transcripts

Adult *Daphnia* (115 inds) were collected and subjected to total RNA extraction according to the above-mentioned procedure. Beforehand, eggs were removed from the brood chamber. Polyadenylated RNA was purified from 500 μg of total RNA using the PolyATtract mRNA Isolation System (Promega Corporation, Tokyo, Japan) and used for 5′ and 3′ Rapid Amplification of cDNA Ends (RACE) using the GeneRacer (Invitrogen, Carlsbad, USA) and SMARTer RACE cDNA Amplification (Clontech Laboratories, Mountain View, WI, USA) kits, respectively. The primers used for cDNA amplification are listed in S1 Table. PCR was performed using KOD+ DNA Polymerase (Toyobo, Osaka, Japan). PCR products were verified by agarose gel electrophoresis, purified, cloned using a Zero Blunt TOPO PCR Cloning Kit (Invitrogen, Carlsbad, USA), and sequenced. The nucleotide sequences were submitted to the DNA Data Bank of Japan (DDBJ) website, with accession numbers LC547945 (*Spo*) and LC547946 (*Jhamt*).

### Quantitative RT-PCR

Embryos were collected at 0, 6, 12, 18, 24, 36, 48, and 72 h after ovulation. These timepoints correspond to specific embryonic developmental landmarks described in [30, 37, 60]. Samples were collected in three biological replicates and subjected to total RNA isolation as described in the preceding section. Synthesis of cDNA was performed using a random primer from 1 μg of the purified total RNA with the SuperScript III Reverse Transcriptase (Invitrogen, Carlsbad, USA). The absence of genomic DNA (gDNA) contamination was confirmed as described previously [61]. PCR was performed in an Mx3005P (Stratagene, La Jolla, CA, USA) instrument using the Power SYBR Green PCR Master Mix (Invitrogen, Carlsbad, USA) with primers listed in S1 Table. PCR amplification was performed in triplicate under the following conditions: 10 min at 95°C, followed by 40 cycles of 95°C for 15 s and 60°C for 1 min. Primer specificity was confirmed by analyzing dissociation curves. Expression levels of each gene were normalized against those of the ribosomal *L32* gene.

### RNAi and microinjection

Small interfering RNAs for *Spo* and *Jhamt* were designed using the Block-iT RNAi designer (http://www.invitrogen.com/rnaidesigner.html). The siRNA sequences were: siRNA-*Spo* (5′ CCGUCUUCUUGCGAUCAAAA 3′); siRNA-*Jhamt*#1 (5′ GGACUUCGGUUGUGGUGAU 3′); siRNA-*Jhamt*#2 (5′ GGCACCAUCUGCAGAUGAA 3′). Two nucleotides dTdT were added to the 3′ end of the siRNA strand. Two additional siRNAs, one containing a random sequence (5′ GGUUAAGCCGCCUCACAU 3′) (siRNA-scrambled) [50], and another one targeting the *Escherichia coli MalE* gene (siRNA-MalE) were used as controls. Microinjection was performed according to an established protocol [29]. Briefly, freshly ovulated eggs from 2–3-week-old *Daphnia* were collected and placed in ice-cold M4 medium [62] containing 80 mM sucrose (M4-Sucrose). Specific siRNA samples for each experiment were mixed with 5 μM AlexaFluor 568 fluorescent dye (Invitrogen, Carlsbad, USA) or Lucifer Yellow dye (Invitrogen, Carlsbad, USA) as an injection marker. After injection, intact eggs were transferred and cultured individually inside 96-well plates filled with 100 μL M4-Sucrose at 23°C. Injected embryos were collected in three biological replicates and subjected to total RNA isolation as described above. Ten micrograms of yeast tRNA (Ambion) were added to each sample as carrier RNA. Synthesis of cDNA was performed using a random primer with a PrimeScript II 1st Strand cDNA Synthesis Kit (TaKaRa, Shiga, Japan). Gene expression levels were evaluated by qPCR using the primer pairs shown in S1 Table.

### Rescue with 20-hydroxyecdysone (20E) and Fenoxycarb (FC)

Shortly after siRNA injection, intact eggs were transferred into 100 μL M4-Sucrose containing 0.01% dimethylformamide (DMF) as control, 1 μM (500 μg/L) 20-hydroxyecdsone (Tokyo Chemical Industry, Tokyo, Japan), or 33 pM (0.01 μg/L) Fenoxycarb (Wako Pure Chemical, Osaka, Japan) in 0.01% DMF. Embryos were incubated at 23°C in the dark. At designated time points, development was observed microscopically.

### Fluorescence photography and image quantification

Fluorescence micrographs of embryos were acquired with a Leica DC500 CCD digital camera mounted on a Leica M165FC fluorescence microscope (Leica Microsystem, Mannheim, Germany). GFP-expressing embryos were imaged using a GFP2 filter.

### Statistical analysis

Differences between datasets were calculated using Student’s *t*-test or Welch’s *t*-test. For multiple comparisons, differences were assessed using Kruskal-Wallis analysis followed by the Dunn post-hoc test. P-values were further adjusted using the Benjamini-Hochberg procedure. All calculations were performed using R 3.6.2 for MacOS software (R Foundation for Statistical Computing).

## Supporting information

S1 Fig Similarities of *D*. *magna* Spo protein to those of other arthropods. (A) Comparison of *Daphnia magna* (Dmagna) Spo protein length with those of *Daphnia pulex* (Dpulex), *Drosophila melanogaster* Spo (DmelSpo), *Drosophila melanogaster* Spookier (DmelSpok), *Tribolium castaneum* (Tcastaneum), and *Bombyx mori* (Bmori). Accession numbers are provided in S2 Table. Percentages indicate identities. (B) Multiple alignment of Dmagna, Dpulex, Dmel (Spo), Dmel (Spok), Tcastaneum, and Bmori Spo proteins. Asterisks, semicolons, and dots indicate conserved, strongly similar, and weakly similar residues, respectively. The positions of characteristic P450 motifs are indicated by black boxes under the red characters.
(TIF)

S2 Fig Similarities of *D*. *magna* Jhamt protein with those of other arthropods. (A) Comparison of *Daphnia magna* (Dmagna) Jhamt protein length with those of *Daphnia pulex* (Dpulex), *Drosophila melanogaster* (Dmel), *Tribolium castaneum* (Tcastaneum), and *Bombyx mori* (Bmori). Percentages indicate identities. Accession numbers are provided in S2 Table. (B) Multiple alignment of Dmagna, Dpulex, Dmel, Tcastaneum, and Bmori Jhamt proteins. Asterisks, semicolons, and dots indicate conserved, strongly similar, and weakly residues, respectively. Putative S-adenosyl-L-methionine (SAM) binding sites are indicated by red boxes.
(TIF)

S1 Table Oligonucleotides used in this study. (DOCX)

S2 Table Accession numbers of proteins used in multiple alignments. (DOCX)
