# Supplementary figures and images for: Roles of and cross-talk between ecdysteroid and sesquiterpenoid pathways in embryogenesis of branchiopod crustacean Daphnia magna
Source: PLoS One. 2020 Oct 9;15(10):e0239893. doi: 10.1371/journal.pone.0239893 (PMC7546464; doi:10.1371/journal.pone.0239893)

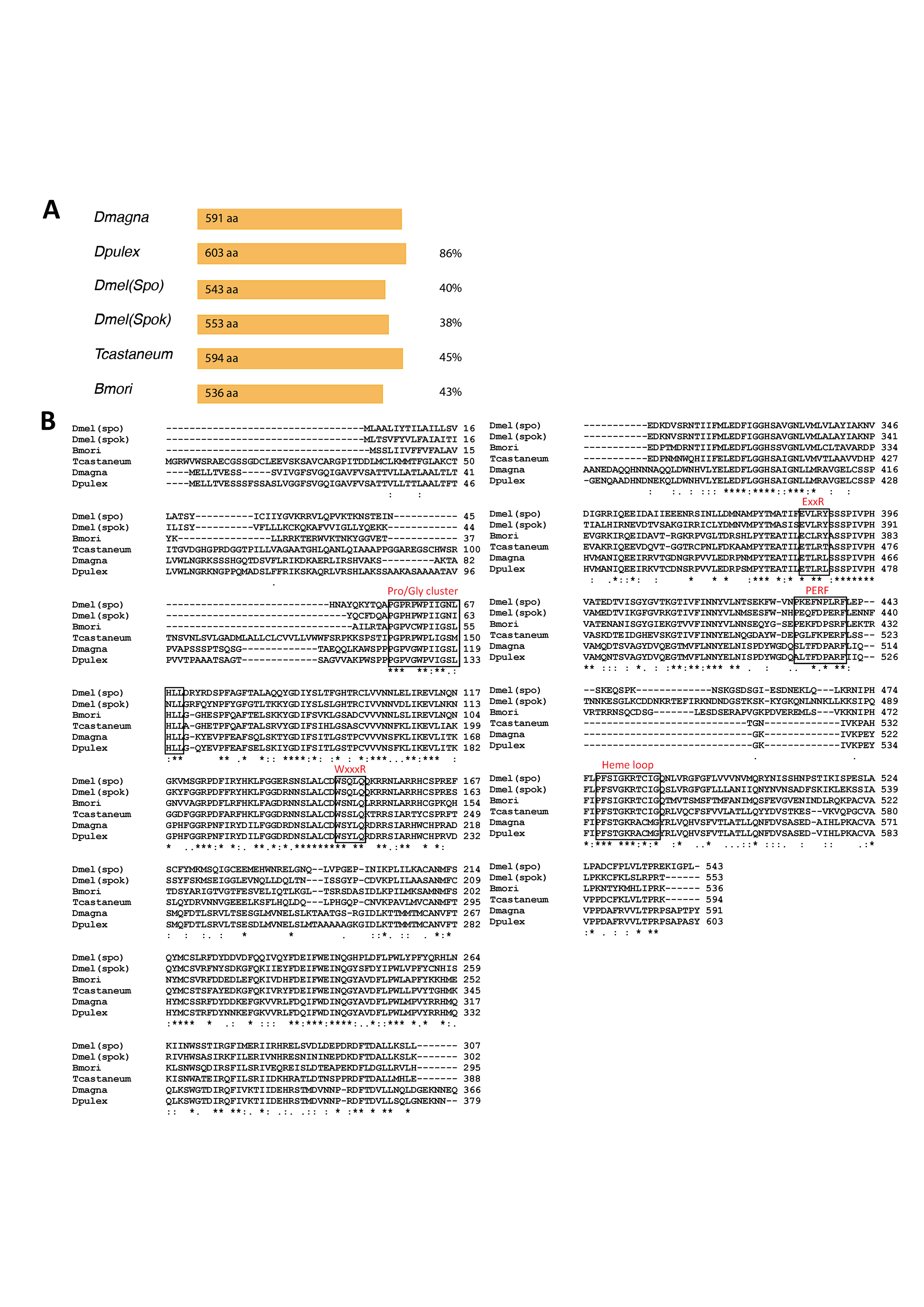

Supplement: S1 Fig — (A) Comparison of Daphnia magna (Dmagna) Spo protein length with those of Daphnia pulex (Dpulex), Drosophila melanogaster Spo (DmelSpo), Drosophila melanogaster Spookier (DmelSpok), Tribolium castaneum (Tcastaneum), and Bombyx mori (Bmori). Accession numbers are provided in S2 Table. Percentages indicate identities. (B) Multiple alignment of Dmagna, Dpulex, Dmel (Spo), Dmel (Spok), Tcastaneum, and Bmori Spo proteins. Asterisks, semicolons, and dots indicate conserved, strongly similar, and weakly similar residues, respectively. The positions of characteristic P450 motifs are indicated by black boxes under the red characters. (TIF) [file pone.0239893.s001.tif]

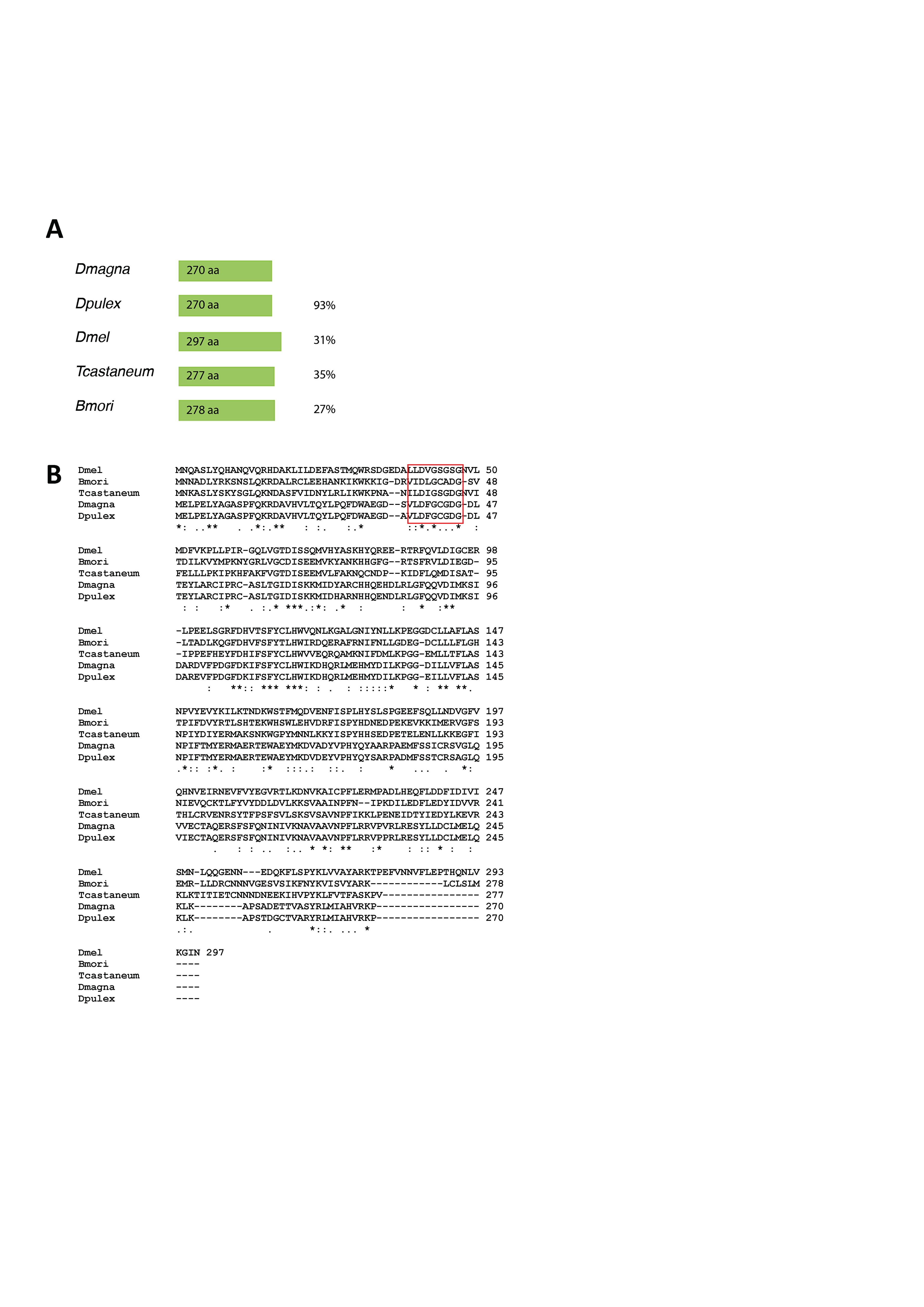

Supplement: S2 Fig — (A) Comparison of Daphnia magna (Dmagna) Jhamt protein length with those of Daphnia pulex (Dpulex), Drosophila melanogaster (Dmel), Tribolium castaneum (Tcastaneum), and Bombyx mori (Bmori). Percentages indicate identities. Accession numbers are provided in S2 Table. (B) Multiple alignment of Dmagna, Dpulex, Dmel, Tcastaneum, and Bmori Jhamt proteins. Asterisks, semicolons, and dots indicate conserved, strongly similar, and weakly residues, respectively. Putative S-adenosyl-L-methionine (SAM) binding sites are indicated by red boxes. (TIF) [file pone.0239893.s002.tif]
