# Supplementary material for: Roles of and cross-talk between ecdysteroid and sesquiterpenoid pathways in embryogenesis of branchiopod crustacean Daphnia magna
Source: PLoS One. 2020 Oct 9;15(10):e0239893. doi: 10.1371/journal.pone.0239893 (PMC7546464; doi:10.1371/journal.pone.0239893)
Supplement: S1 Table — (DOCX) [file pone.0239893.s003.docx]

**S1 Table: Oligonucleotides used in this study**

| **Primers for qRT-PCR** | | | |
| --- | --- | --- | --- |
| **Gene Name** | **Forward (5**′ **– 3**′**)** | **Reverse (5**′ **– 3**′**)** |  |
| *Spo* | GGGCTATGCTGTCGATTTCC | TTGTGCTGTTGTGCGTCTTC |  |
| *Jhamt* | GTGGGCCGAATACATGAAGG | ACGAAGGAACGGGTTGACAG |  |
| *Met* | CGGGTCGTTTGATTTTCCTTC | TCCTTCATTCCTTGCTCTTCG |  |
| *Nvd1* | AGCACAAGGCGGGAAGAGT | GCTTCCCATTTCACCTTCCA |  |
| *Nvd2* | CGTCGGTGACTGCATCGA | TGCCGTCGTTCCCATTG |  |
| *Phm* | CGTGGCTGACAAAACGAACA | CCACCAATCCAAGCAGAAGA |  |
| *Dib* | CCAACGAACGGACCTGAATG | TCGAACGTCACCAAACCAAG |  |
| *Sad* | GCTCCTTCCTCCCTCTTTGG | CGATGCGCTGAATGTCAACT |  |
| *Shd* | GACTGCTGAAGGCGTTGACA | CGGCTGCCACTAGGTCGATA |  |
| *L32* |  |  |  |
| **Primers for RACE** | | | |
| **Primer Name** | **Forward (5**′ **– 3**′**)** | **Reverse (5**′ **– 3**′**)** |  |
| Spo_5RACE-GSP |  | TTCGTTCACCATCAAGCCGCTCTC |  |
| Spo_5RACE-GSP-nested |  | GAACGACGATCCCTCTGCAAGTAG |  |
| Spo_3RACE-GSP | GCCCTACACCGAAGCGACTATTCTC |  |  |
| Spo_3RACE-GSP-nested | TCCGGCTCGATTTCTTATCCAAGG |  |  |
| Jhamt_5RACE-GSP |  | TCCGTTCTGCCATGCGTTCGTAC |  |
| Jhamt_5RACE-GSP-nested |  | CCGTTGATGGTCTTTGATCCAG |  |
| Jhamt_3RACE-GSP | CCACTGGATCAAAGACCATCAACG |  |  |
| Jhamt_3RACE-GSP-nested | CATGTACGAACGCATGGCAGAAC |  |  |
