# Supplementary material for: Roles of and cross-talk between ecdysteroid and sesquiterpenoid pathways in embryogenesis of branchiopod crustacean Daphnia magna
Source: PLoS One. 2020 Oct 9;15(10):e0239893. doi: 10.1371/journal.pone.0239893 (PMC7546464; doi:10.1371/journal.pone.0239893)
Supplement: S2 Table — (DOCX) [file pone.0239893.s004.docx]

**S2 Table: Accession number of proteins used for multiple alignment analysis**

| **Protein Name** | **Species name** | **GenBank accession number** |
| --- | --- | --- |
| Spook | *Daphnia magna* | BCF86811 (this work) |
|  | *Daphnia pulex* | EFX88041 |
|  | *Drosophila melanogaster* | NP_647975 |
|  | *Tribolium castaneum* | EFA11558 |
|  | *Bombyx mori* | NP_001104833 |
| Spookier | *Drosophila melanogaster* | NP_001104460 |
| Jhamt | *Daphnia magna* | BCF86812 (this work) |
|  | *Daphnia pulex* | EFX90188 |
|  | *Drosophila melanogaster* | NP_609793 |
|  | *Tribolium castaneum* | NP_001120783 |
|  | *Bombyx mori* | AB113578 |
